# Adaptive Data Acquisition with Energy Efficiency and Critical-Sensing Guarantee for Wireless Sensor Networks

**DOI:** 10.3390/s19122654

**Published:** 2019-06-12

**Authors:** Yuan Rao, Gang Zhao, Wen Wang, Jingyao Zhang, Zhaohui Jiang, Ruchuan Wang

**Affiliations:** 1 School of Information and Computer Sciences, Anhui Agricultural University, Hefei 230036, China; zhaoweifree@foxmail.com (G.Z.); wangwen@ahau.edu.cn (W.W.); zjy990228@163.com (J.Z.); jiangzh@ahau.edu.cn (Z.J.); 2 Key Laboratory of Agricultural IoT, Ministry of Agriculture Rural Affairs, Yangling 712100, China; 3 Jiangsu High Technology Research Key Laboratory for Wireless Sensor Networks, Nanjing 210003, China; wangrc@njupt.edu.cn

**Keywords:** adaptive, data acquisition, energy efficiency, critical events, sensing guarantee

## Abstract

Due to the limited energy budget, great efforts have been made to improve energy efficiency for wireless sensor networks. The advantage of compressed sensing is that it saves energy because of its sparse sampling; however, it suffers inherent shortcomings in relation to timely data acquisition. In contrast, prediction-based approaches are able to offer timely data acquisition, but the overhead of frequent model synchronization and data sampling weakens the gain in the data reduction. The integration of compressed sensing and prediction-based approaches is one promising data acquisition scheme for the suppression of data transmission, as well as timely collection of critical data, but it is challenging to adaptively and effectively conduct appropriate switching between the two aforementioned data gathering modes. Taking into account the characteristics of data gathering modes and monitored data, this research focuses on several key issues, such as integration framework, adaptive deviation tolerance, and adaptive switching mechanism of data gathering modes. In particular, the adaptive deviation tolerance is proposed for improving the flexibility of data acquisition scheme. The adaptive switching mechanism aims at overcoming the drawbacks in the traditional method that fails to effectively react to the phenomena change unless the sampling frequency is sufficiently high. Through experiments, it is demonstrated that the proposed scheme has good flexibility and scalability, and is capable of simultaneously achieving good energy efficiency and high-quality sensing of critical events.

## 1. Introduction

Wireless sensor networks (WSNs) have emerged as highly flexible and dynamic facets that are being deployed in many applications [1]. With various types of sensors, WSNs can be used in a harsh environment to periodically measure a wide variety of parameters around its surroundings, for example, air temperature, humidity, lightning condition, and forward sampling data to the Sink [2]. In the past decades, due to their increasing capabilities to sense, store, process, and communicate information at low cost, WSNs have continuously gained attention in their potential applications, such as environment monitoring, health care monitoring, agricultural production management, and intelligence surveillance [3,4,5]. However, energy consumption is still a limiting factor for data collection in WSNs because the sensor nodes are typically battery-powered and characterized by a limited energy budget. The main reason is that it is difficult—or even unfeasible—to recharge sensors in many applications [6]. As a result, the inherent restriction of energy carried within the battery of sensor nodes brings an extreme difficulty in obtaining a satisfactory network lifetime [7]. A tremendous number of algorithms and schemes have been proposed with the aim of improving WSNs lifetime [8,9]. As pointed out in [10], it is well-known that the biggest energy drain should be ascribed to the communication module. This is unfortunate, given that the ability to gather data is the one motivating the use of WSNs in many pervasive computing applications [8].

In the context of continuous monitoring, most data changes at a slow speed, which causes a large amount of data redundancy in space or time, whereas frequent data transmission among sensor nodes would be a waste of limited energy. Basically, the increase of network lifetime will be proportional to the reduction in the number of transmitted data packets. As a result, in order to limit energy consumption, data reduction without compromising data quality has become one of the most popular strategies, with methods including information encoding, data aggregation, data compression and their combination [11]. As for data compression, there are mainly two kinds of typical modes: Compressed sensing (CS) and prediction-based approaches. In the latter, the Sink has the same data prediction model as sensor node. Data prediction is, in parallel, implemented at both sensor nodes and the Sink. As long as the locally sensed data are compatible with the model prediction, no further communication is needed: Only when the sensed data differs by more than certain threshold from the model must the new model be trained and synchronized. The CS allows a sparse analog signal to be represented by much fewer samples than that required by the Nyquist sampling theorem [12]. In other words, for a sparse signal, only a small number of encoded samples, generated by using the original samples and properly chosen transform coefficients, are needed to be reported and used to recover the original signal at the Sink. In the past years, the CS has been regarded as an effective paradigm for achieving high sensing quality at a low sampling rate. 

Unfortunately, sparse sampling of the signal makes it hard to obtain the incomplete data in real time because data reconstruction is required, but is usually conducted later. This leads to the fact that it is difficult for sensing important events in time when the data trace goes into the critical zone to be monitored. As a matter of fact, this problem could be alleviated or even solved if using prediction-based methods. On the other hand, the model synchronization incurs extra in-node computation and communication overhead in prediction-based approaches. Therefore, their practical applicability remains unclear. Based on the characteristics of the two data gathering modes, one can presume that it is a promising means for further improving energy efficiency, as well as sensitivity to sensing critical events, to integrate CS with prediction-based approaches. Intuitively, the integration has potential for reducing the frequency of data sampling and the amount of data transmission in the case of non-critical data zones, and increasing the sampling frequency for timely data acquisition in the case of critical data zones. However, the combination method in [13] has difficulties in reducing the sampling frequency and computation overhead, and dealing with effective switching between two data gathering modes.

In order to handle the aforementioned difficulties, one adaptive data acquisition scheme is proposed with the goal of simultaneously achieving good energy efficiency and timely sensing of critical events in WSNs. The contribution of this work is as follows. First, one framework is developed for the integration of CS and prediction-based approaches. Secondly, one setting strategy of adaptive deviation tolerance is proposed for improving the flexibility of the proposed scheme. Thirdly, an adaptive switching mechanism of data gathering modes is put forward for overcoming the drawbacks in the traditional method that fails to quickly react to phenomena change unless the sampling rate is sufficiently high. Finally, experiments are performed based on a realistic data trace to validate the performance of the proposed scheme. Some suggestions are made for guiding the integration of CS and prediction-based approaches to effectively deal with the acquisition of monitored data.

The remainder of this paper is organized as follows: In Section 2, we present a brief overview of previous work about suppressing data transmission for WSNs. Section 3 explains our data acquisition scheme, as well as adaptive deviation tolerance and switching mechanism. The experimental results and discussion are presented in Section 4. Finally, conclusions and further research are addressed in Section 5.

## 2. Related Work and Motivation

For the purpose of improving energy efficiency, the reduction of data transmission amount has become one of the most popular strategies among data gathering methods for WSNs. The typical way of obtaining data reduction is to use sparse sampling or replace the real value with the predicted value. In the past decades, the CS and prediction-based approaches have been adopted as two representative techniques of data reduction.

### 2.1. Compressed Sensing Approaches

First, the basic idea of compressed sensing is presented. Consider a discrete signal denoted by vector $x_{N\times 1}$. If x is sparse enough, that is, $K=||x|{|}_{0}\ll N$, it is possible to reconstruct x from *M* measurements produced by a proper linear transform Φ [14,15]:

(1) $$y_{M\times 1}=\Phi_{M\times N}x_{N\times 1}$$

Here, K<M≪N, M/N is referred to as the sampling rate, Φ is usually termed as measurement matrix. However, in practice, most signals may not be truly sparse or sufficiently sparse. Nevertheless, it can be sparsely represented in an alternative domain, that is, $x_{N\times 1}=\Psi_{N\times N}\alpha_{N\times 1}$, where Ψ is referred to as the representation basis and α is sparse in Ψ domain. The CS formula can be expressed as

(2) $$y_{M\times 1}=\Phi_{M\times N}\Psi_{N\times N}\alpha_{N\times 1}$$

As the number of measurements *M* is much less than the number of original samples *N*, it is an under-determined linear system. This ill-conditioned system can be solved by minimizing the $l_{0}$ norm. However, it is NP-hard if directly solving the problem. Fortunately, when ΦΨ satisfies the restricted isometric property (RIP), there is an alternative kind of approach seeking to solve the $l_{1}$ norm minimization problem instead of the $l_{0}$ norm-based problem [15]. Therefore, we have

(3) $$\hat{a}=\underset{\tilde{\alpha }\in R^{N}}{\mathrm{argmin}}||\tilde{\alpha }|{|}_{1},\;s.t.\;y_{M\times 1}=\Phi_{M\times N}\Psi_{N\times N}\alpha_{N\times 1}$$

where $\tilde{\alpha }$ is the recovered signal in the representation basis. The problem can be easily solved using existing recovery algorithms. Once determining $\hat{a}$, the corresponding signal $\hat{x}$, an approximation of the original signal, can be obtained as

(4) $${\hat{x}}_{N\times 1}=\Psi_{N\times N}{\hat{a}}_{N\times 1}$$

Assume that there is one chain network with N sensor nodes and one Sink located at the end of the chain. Each node collects or generates data packets $x_{i},i=1,\cdots ,N$. Considering CS-based data acquisition model formulated in Formulas (1)–(4), the N raw packets can be represented by M packets (far less than N) and then recovered at the Sink. During the process of its transmission, each raw packet $x_{i}$ is weighted by $\vec{\phi_{j}}$, where $\vec{\phi_{j}}$ is the *j*-th column of measurement matrix $\Phi_{M\times N}$, 1≤j≤N. As a result, the Sink receives M weighted data packets and thus only MN times of transmissions in total occur, and all network nodes consume the same amount of energy for data transmissions. In contrast, if using the traditional data acquisition method, the sink receives N raw packets, N(N+1)/2 transmissions are needed, and the closer one node is to the Sink, the more energy consumption it will suffer from. Additionally, the CS has tolerance to transmission losses/failures, since only a sufficient number of packets are needed for data recovery [14]. Therefore, the CS-based data acquisition has significant advantages in terms of energy consumption balance, energy efficiency, and transmission robustness. In the past decades, there has been increasing attention on CS-based compressed data gathering (CDG-CS) in WSNs, for improving energy efficiency and network throughput. To recover the original data with high accuracy, three components, namely, sparse basis, measurement matrix, and reconstruction algorithm, often need to vary with the specific application data [12,16,17].

As a matter of fact, the measurement matrix plays a vital role in reducing the amount of data transmission for WSNs. Among applying CS to the data gathering, a lot of work has attempted to design the measurement matrix by taking into consideration the characteristics of WSNs. According to their design idea, the main methods of CDG-CS can be divided into the following categories: Sparse projection, data transmission model and their combination. When adopting the dense measurement matrix, a large number of sensor nodes would get involved for each CS measurement, leading to inefficient energy consumption in WSNs [14]. To solve this problem, sparse projection including lots of “0” elements is proposed, which means that the sensor nodes corresponding to “0” element do not need to conduct data gathering during each round of data measurement and transmission. As a result, this helps with energy conservation because the number of involved sensor nodes is significantly reduced. In [18], an energy efficient data aggregation scheme for a weighted sensor model is proposed by jointly considering the sparse sampling and the power control ability in sensor nodes. It is confirmed that the sparse random measurement matrix has advantages of reducing the energy consumption. In [19], considering the relation between the sparse binary matrix and data gathering, one sparse binary matrix is chosen as the measurement matrix for addressing the energy balance problem of compressed data gathering in WSNs.

In order to further suppress data transmission, some authors attempt to enhance the CDG-CS schemes by considering data transmission model in WSNs. Following this idea, the integration between CS and random walk (RW) routing is proposed for reducing energy consumption of WSNs [20]. In this work, each CS measurement is collected through a RW routing with a predefined length. All random CS measurements are forwarded to the Sink for the CS recovery process in either of two either: Directly or by relaying through intermediate nodes. The trade-off between the sensor transmission range and the length of RWs is investigated for the networks to achieve the smallest energy consumption. Another method is the integration of CS and spanning tree routing for data compression. It was shown that the hybrid CS can achieve significant improvement in terms of throughput, since it avoids the excessive traffic load at leaf nodes and also takes advantage of CS to reduce the traffic load at those nodes near the Sink [13]. In [21], the authors present an integration of CS and clustering in WSNs, utilizing block diagonal matrices as the measurement matrices. In [22], the network topology is first converted into a logical chain, and then the spatial correlation of data within the same cluster is employed for CS. It has been argued that compared with CS, which is based on tree topology, the compressed data gathering methods based on clustered topology are capable of offering higher recovery quality and compression ratio. In [23], the proposed schemes selects different nodes at random as projection nodes, and sets each projection node as a root to construct a minimum spanning tree by combining with interest nodes. It is the projection node that takes charge of aggregating data from sensor nodes using CS approaches. 

As presented above, the CDG-CS has made great breakthroughs in recent years. However, for data gathering applications, most existing CDG-CS methods adopted aim at increasing the overall networking efficiency. It comes to our attention that it is difficult to apply these existing CDG-CS methods to implement data gathering in small-scale sensor networks where signal sparsity may not be prominent enough and hence the potential capacity gain will be very limited. Moreover, due to the constraint of sensor cost, many applications have to face the low-density deployment of sensor nodes, which would cause a lack in the signal sparsity, limiting the practicability of the existing CDG-CS. Therefore, some authors have paid attention to the research on the sparse sampling and reconstruction of the target parameters from the perspective of a single node using CS. In [24], the problem of reconstructing the soil moisture process is considered at a single location. The authors construct a sparse basis by exploiting unique features of soil moisture evolution and show that this basis attains a very good trade-off between its ability to sparsify the signal and its incoherence with measurement matrices. Furthermore, in order to minimize the number of data transmission with satisfactory reconstruction quality, the dynamic sampling schedule is put forward by taking advantage of the prior knowledge obtained by means of analysis on the earlier measurement data [13,25,26].

### 2.2. Prediction-Based Approaches

Essentially, the gathered data in WSNs is a sequence of data points, typically consisting of observations made over a time interval and ordered in time. This makes it possible to use a time series as an input to make predictions if there was one appropriate prediction model [10]. Indeed, there has been much work and discussion concerning data prediction in various application domains of WSNs. This method involves training models that could be used to predict future data traces, either at the Sink or on the nodes. Most of the prediction models, approximating or predicting sensor measurement, generally take the following form:

(5) $$\{\begin{array}{l} u_{t}={\left[v_{t-1},v_{t-2},\cdots ,v_{t-L}^{}\right]}^{T}\\ \;\varepsilon_{t}=|v_{t}-f(u_{t})| \end{array}$$

where $u_{t}$ is the training set, denoting the historical value obtained at time t; *L* is the length of the training set; $v_{t}$ is the sensor measurement, which could be temperature, illumination, humidity, etc.; f(u) is a data prediction model trained using the historical data training set; and $\varepsilon_{t}^{}$ is the prediction error.

When conducing prediction-based approaches for data acquisition, the Sink and sensor node simultaneously use the same prediction model to make its own data prediction. After the sensor node compares the predicted values with those actually observed, no further action is required if the prediction error is within the deviation tolerance. Subsequently, the Sink directly takes the prediction value as the collected value. Otherwise, the data collected by the sensor need to be sent to the Sink. Subsequently, the prediction model is re-trained based on historical data and synchronized between the Sink and sensor nodes. As in (5), these predictions are represented as a function of the past observations and their respective times. The prediction models that have been proposed can be split into three classes: Time series modeling, regression methods, and machine-learning techniques [10].

Generally, the time series modeling includes autoregressive (AR), moving average (MA), autoregressive integrated moving average (ARIMA), and exponential smoothing (ES). The AR term uses the linear regression function of prior values to calculate the estimation of future sensed data. The MA method calculates the weighted average of the observations recently made. Incorporating the AR, integrated, and MA term, ARIMA is powerful for analyzing and building the forecasting model which best represents a time series by modeling the correlations within the available historical data [10]. Among time series modeling methods, ARIMA is broadly used in different applications, such as sea-surface wind speed, air temperature and humidity, and electricity demand forecasting [8,27]. It has been argued that ARIMA is able to capture a wide variety of realistic phenomena with inexpensive memory and computational overhead. The ES continually revises a forecast in light of more recent observation by assigning exponentially decreasing weights as the observation gets older [27]. 

As the simplest kind of regression, linear regressions are used to characterize linear relations between the observed variables. DBP (derivative-based prediction), proposed in [28], is one typical example of linear regression. The model is built based on the first and last few edge points within the learning window. The idea behind DBP is to build one simple model that can effectively capture the trends in recently-observed data, instead of reducing the approximation error, which enables DBP be resilient to the noise inherent in the data. Two typical machine-learning techniques, namely, artificial neural networks (ANNs) and support vector regression (SVR), have been applied for suppressing data transmission [10,29]. Particularly, the new generation of ANN, clockwork recurrent neural network, has been investigated for data prediction in WSNs [30]. However, machine-learning models require more computational resources from the sensor node [10].

The prediction-based methods are able to guarantee the timely data acquisition, as well as the suppression of data transmission. Theoretically, high accuracy forecasts can reduce the amount of transmissions by 30–80% (varying with application cases) with satisfactory accuracy [31,32]. However, it is required for the operation mode to build a model and to transmit the model parameters among the network. The model build-up process might be computationally expensive for a node to implement, especially due to memory limitation. Since the monitored data may change with time, the parameters used to define a model may become obsolete after a while [10]. Therefore, there is a computational and communication cost to re-train and synchronize the parameters of the chosen method and maintain the prediction’s accuracy. Additionally, when performing each prediction, the sensor node needs to collect data and compare these with the predicted data to determine whether data transmission is needed. These are additional computation overheads that cannot be ignored.

### 2.3. Summary and Motivation

In summary, there already exists some work concentrating on the CS-based compressed data acquisition of a single sensor node. Since the theory of CS is based on sparse sampling, the sensor nodes are only awakened when sampling, and others are in a sleep state, which leads to the inability to obtain the monitored data in time. Although the prediction algorithm can provide the timely data acquisition, it inevitably needs to conduct data sensing, data prediction, model training and synchronization during the data collection, which leads to a large amount of additional expenses. Therefore, there exists complementarity between the CS- and prediction-based approaches. One good attempt, called SWIFTNET, has been made in [13]. This work aims at achieving compressed data acquisition with high reactiveness, aggressive data collection and transmission for wildfire monitoring application by one combination of data compression, prediction and adaptive sampling strategies. However, there are still some open issues that are worth further investigating. In SWIFTNET, when the CS is implemented, the sensor maintains the original sampling rate and the data transmitted to the Sink are obtained by calculation. This operation only suppresses the data transmission but leaves the frequent sampling, introducing an additional calculation overhead, which is particularly undesirable for wireless networks. This means that there might be an opportunity for further reducing energy consumption. Secondly, one predefined error bound, ±0.3 °C, is used for controlling whether data are transmitted or suppressed. Such an arbitrarily predefined deviation tolerance might be an obstacle to flexibility.

Intuitively, when implementing CS, it would definitely contribute to reducing energy consumption if the sampling frequency deceased, leading to the sparse distribution of data points. As a result, it is challenging to develop one effective switching mechanism from CS to prediction-based modes. It should be emphasized that in many monitoring applications, there might be two or even multiple critical zones with various levels of urgency. For example, in some applications (e.g., agricultural breeding and nursery), it is demanded that the favorite environmental parameters are within one range, so that beyond the range there are more critical zones (further explanation in Section 3.3). On the other hand, there are differences in the values and fluctuation characteristics of monitored data. Hence, when using arbitrarily predefined deviation tolerances, the specific tolerance value is supposed to change from one dataset to another. Consequently, the deviation tolerance has to be manually adjusted through the analysis of a dataset’s characteristics, which is time- and labor- consuming. It would be helpful for improving the flexibility if the deviation tolerance was adaptive. The main goal of this paper is to achieve adaptive data acquisition with energy efficiency and high-quality sensing of critical events, by means of exploring the adaptive deviation tolerance and switching mechanism between CS and prediction-based modes, especially under the condition of sparse distribution of sampled data points. Compared with SWIFTNET, the proposed scheme has the characteristics of much lower sampling frequency, better flexibility and sensing ability of critical events by overcoming the shortcomings of arbitrarily predefined deviation tolerances and switching mechanisms between CS and prediction-based modes. Additionally, comparisons are made in terms of data acquisition accuracy and compression ratio, to develop suggestions for the further integration of prediction-based approaches into data acquisition schemes.

## 3. Adaptive Data Acquisition Scheme

In this section, one integration framework, taking respective advantages of compressed sensing and prediction-based approaches, is designed for developing the adaptive data acquisition scheme. Afterwards, we investigate two key techniques: Adaptive deviation tolerance and adaptive switching mechanism of data gathering modes.

### 3.1. Data Acquisition Framework

**Figure 1:**
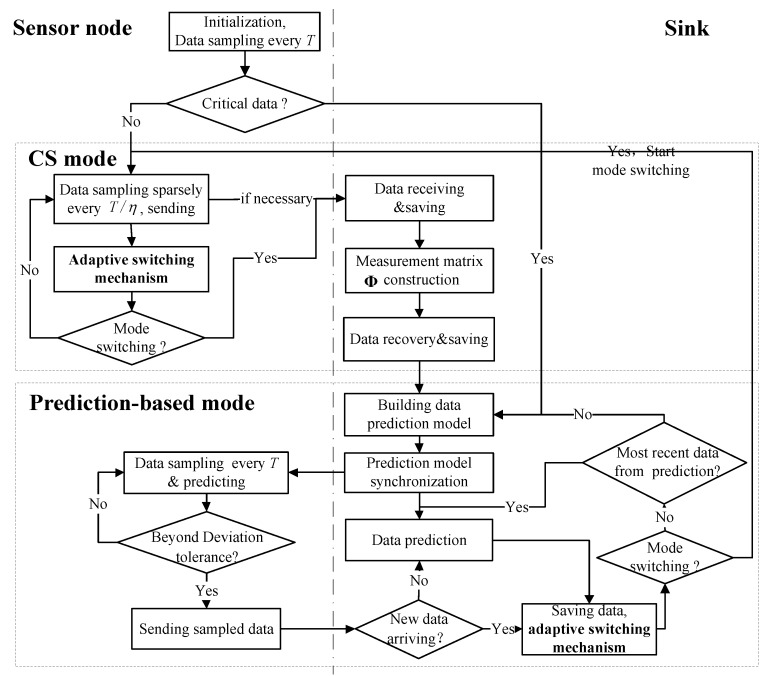
Framework of adaptive data acquisition scheme.

Figure 1 depicts a framework of the proposed scheme, which explicitly shows the way the integration of CS and prediction-based modes works. During data collection, the proposed scheme intelligently activates one of two modes. More specifically, CS works for the acquisition of non-critical data, while prediction-based methods are in charge of the acquisition of critical data. It is important that there is one appropriate switching mechanism between CS and prediction-based modes, which is presented in detail in Section 3.3. One or multiple prior thresholds, predefined depending on the specific applications, are used for identifying critical and non-critical data traces.

At the beginning of data acquisition, the sensor node takes several readings at the original time interval, *T*. Afterwards, the sensor node determines whether the currently sensed data trace is critical or non-critical by checking the relation between their average and prior thresholds; in essence, dependently on the requirement of specific applications. If the current data are critical, the CS is activated for gathering data; subsequently, sparse sampling is implemented. Otherwise, the prediction-based approach is activated instead, and it triggers the training of data prediction model and timely acquisition of the interested phenomenon.

When entering the data prediction mode, the sensor node takes readings at the original time interval, *T*. In our study, the Sink is assumed to be rich in energy-resource due to unlimited power supply, and it is capable of performing model training. One model of the data trend can be trained based on the historical data at the Sink and then the model parameters are transmitted to the sensor nodes. Afterwards, dual data prediction is implemented at the Sink and on the sensor node using the same model. As long as the locally sensed data are compatible with the model prediction, no further communication is needed. Only when the sensed data deviates from the model, the collected true value is transmitted to the Sink to re-train and synchronize the data prediction model. Assume that the Sink can synchronize with the sensor nodes at each time stamp. For the missed value of the same sampling moment as the sensor nodes at the Sink, the prediction value is directly used as the collected value, instead of raw data. Additionally, during implementing data prediction modes, at every original sampling time interval the Sink invokes the adaptive switching mechanism to check if it meets the condition of activating CS. If the condition is satisfied, the Sink and sensor node are notified of activating CS. Otherwise, one of the following two actions are implemented: (a) The Sink re-trains and synchronizes the prediction model if the latest data are from raw data; (b) the Sink and sensor node proceed with data prediction using the previous prediction model. 

When the CS mode is activated, the sensor node takes readings in a sparse sampling way, at the time interval of T/η, where η denotes sampling rate that is the ratio between total amount of compressed data and the total amount of original uncompressed data. In this way, the node energy consumption is effectively reduced, since the sensors are powered off when there are no readings taken. Subsequently, the sensor node invokes the adaptive switching mechanism to check whether the mode switching condition is satisfied after the sensor node conducts every round of sparse sampling. If the condition is satisfied, the sensor node notifies the Sink of reconstructing the data and activating prediction-based mode. Otherwise, the Sink and sensor node continue implementing CS.

Given *M* sparsely sampled data points, the Sink constructs the measurement matrix $\Phi_{M\times M/\eta }$ with the size of M×(M/η), in which the elements are equal to zero except that those elements with the location of (i,(i/η)) equal one. Here, the symbol i denotes the row number in matrix $\Phi_{M\times M/\eta }$, numbered from 0, and i∈[0,M−1]. In our case, there is no need to transmit the measurement matrix for scheduling data acquisition, which helps in avoiding a potential large burden. The difference matrix proposed in [24] is used to construct sparse matrix $\Psi_{M/\eta \times M/\eta }$. Smoothed L0 (SL0) is recommended as the reconstruction algorithm. Therefore, the original data can be reconstructed according to Formula (3) and (4).

### 3.2. Adaptive Deviation Tolerance

As pointed out in Section 2.2, one deviation tolerance is required for determining when to re-train and synchronize the prediction model during implementation of the prediction-based approach. It would help improve the flexibility of the proposed scheme if the deviation tolerance could dynamically adapt to the monitored data. Note that, for long-term monitoring applications, the phenomena tend to have inherent characteristics of continuous change. This offers one possibility of determining current deviation tolerance, by taking into account the characteristics of historical data.

Here, one adaptive deviation tolerance is proposed based on the previous gathering data and Student’s t-distribution. Firstly, the data trace from the previous day is evenly divided into 24 segments. Then, the prediction deviation tolerance (PDT) is calculated according to Student’s t-distribution [29]. The PDT gives the range of deviation values that the prediction can take, and the prediction level (q=100(1−θ)) indicates the expectation of the prediction deviation within the PDT. If it is assumed that the model residuals have a zero-mean Gaussian distribution, the %q PDT for each segment can be calculated as

(6) $$d_{i}=t_{\theta /2,n_{i}-1}\times S_{i}\sqrt{1+\frac{1}{n_{i}}}$$

where $t_{\theta /2,n-1}$ is the *q*-th percentile of a Student’s t-distribution with $n_{i}-1$ degrees of freedom; θ is the significant level; and $S_{i}$ and $n_{i}$ are the standard deviation and the number of the considered data within *i*-th section, respectively. 

Finally, one can get the average prediction deviation tolerance (APDT) as follows:

(7) $$d_{avg}=\frac{\sum d_{i}}{24},\;i=1,2,\ldots ,24$$

The APDT provides a reference for determining whether there is requirement for re-training and synchronizing the model. That is to say, during implementing prediction-based approach, a prediction range (PR) is set based on the sensed true data and average prediction deviation tolerance:

(8) $$PR=v_{true}\pm d_{avg}$$

where $v_{ture}$ is the true data sensed from the sensor. If the new prediction falls within the bounds of the PR, then the model is regarded as appropriate. Otherwise, it is time to conduct re-training and synchronization of the data prediction model. 

The benefit of using the prediction deviation tolerance instead of an arbitrary threshold is that the prediction level guides the selection of the prediction range. Therefore, one can adjust the prediction deviation tolerance by choosing different significant levels according to the specific application. It should be noted that the data from the previous day could be divided into greater or fewer segments in practice.

### 3.3. Adaptive Switching Mechanism

For the purpose of good energy efficiency and high-quality sensing of critical events, we focus on how to adaptively and effectively conduct switching between CS and prediction-based modes within the aforementioned integration scheme of data acquisition. Note that there are two switching scenarios that is, switching from CS to prediction-based modes and vice versa. Particularly, due to the sparse distribution of sampled data points, it is challenging to determine the switching moments from CS to prediction-based modes. Moreover, it is an essential part for developing appropriate switching mechanism to take into account good sensitivity to critical data trace, as well as avoidance of unnecessary switching. The main idea is comprehensively explained in this section, where the adaptive switching mechanism from prediction-based to CS modes is firstly described, followed by the opposite switching mechanism.

**Figure 2:**
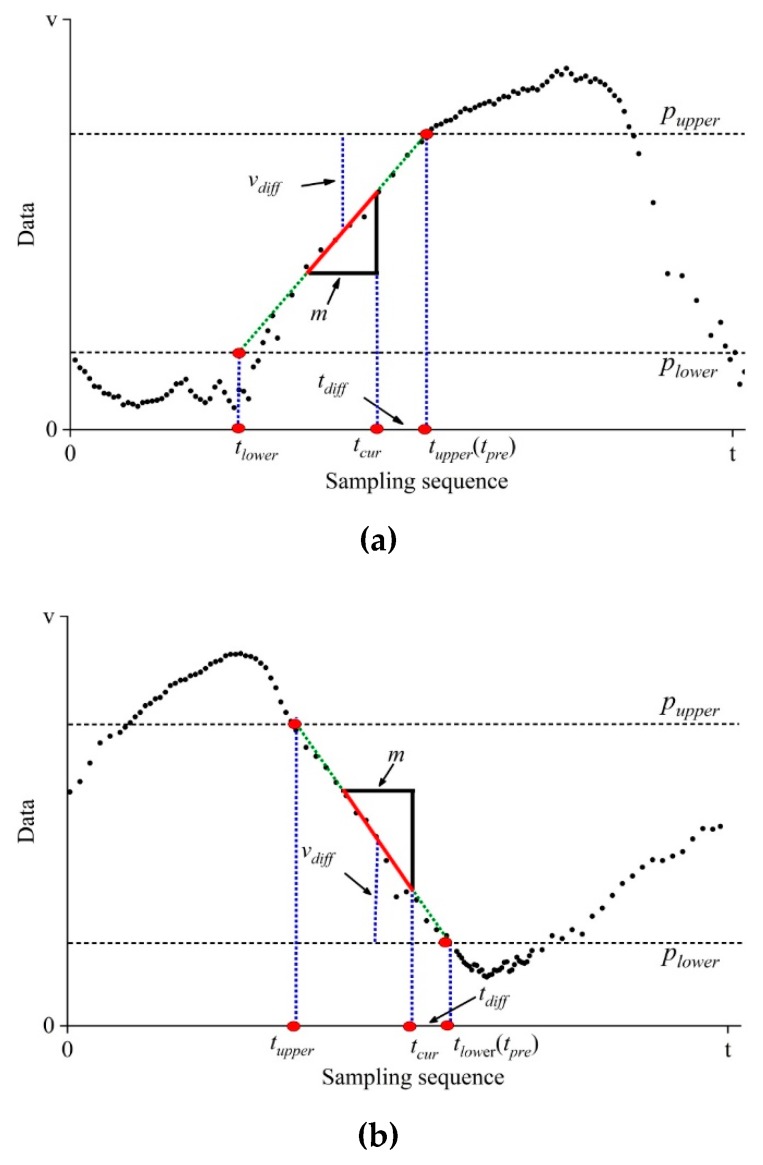
Two switching cases from CS to prediction-based modes: (**a**) Data trace across upper threshold. Data trace exhibits increasing trend, and switching behavior occurs in the vicinity of sparsely sampled data approximating upper threshold; (**b**) Data trace across lower threshold. Data trace exhibits decreasing trend, and switching behavior occurs in the vicinity of data approximating lower threshold.

Here, double prior thresholds of $p_{upper}$ and $p_{lower}$ are defined, for identifying critical and non-critical data trace, since it can be easily extended to the applications with one or multiple prior thresholds. As shown in Figure 2, assume that data beyond the range $\left[p_{lower},p_{upper}\right]$ are critical; thus the prediction-based mode is required for timely data gathering. On the contrary, the CS is responsible for gathering non-critical data within the range $\left[p_{lower},p_{upper}\right]$.

Regarding the switching from prediction-based to CS modes, the number of continuous data points is considered. Although there are more data points in prediction-based modes compared with CS, it still has potential to suffer from the frequent change of data gathering modes caused by data fluctuation around the prior thresholds. For the purpose of efficiently monitoring the critical information, the CS does not go back for data gathering until the average of these data is beyond the range $\left[p_{lower}+d_{avg},p_{upper}-d_{avg}\right]$. This discriminative range enables the switching mechanism to be tolerant to the data fluctuation around the prior thresholds, which helps in avoiding the unnecessary changing of data gathering modes, as well as offering a guarantee for the collection of critical information.

As for the switching from CS to prediction-based modes, two typical cases are illustrated in Figure 2. When implementing CS, the sensor node sparsely takes readings at the time interval of T/η, where η denotes sampling rate. After taking readings every time, the sensor node immediately calculates the average value of the most recent *m* data points as follows,

(9) $$\bar{v}=\sum_{i=1}^{m}\frac{v_{i}}{m}$$

Then, the difference $v_{diff}$ between the average of *m* data points and the prior thresholds is calculated,

(10) $$v_{diff}=\min\left(\left|\bar{v}-p_{upper}\right|,\left|\bar{v}-p_{lower}\right|\right)$$

Suppose that we have the discriminative threshold, $v_{tol}$ for the difference of sampling values. Subsequently, if $v_{diff}$ is less than $v_{tol}$, the sensor node continues implementing CS at the next sparse sampling moment. Otherwise, the sensor node proceeds with the subsequent procedure. 

In order to build the estimation model of switching moment, the sensor node performs line fitting using the most recent *m* data points as follows,

(11) $$\hat{v}\left(t\right)=\alpha t+\beta$$

It should be pointed out that one discrete numeric value, t, denotes the sampling sequence when data gathering is performed at the original interval *T*. In order to make it helpful for understanding, we use the term moment instead of the sampling sequence in this section. The symbol α denotes the change trend of data trace, β is intercept.

Mathematically, the estimated switching moment is predicted by the intersections between the prior thresholds and fitting line of *m* data points as follows,

(12) $$t_{pre}=\left\{ \begin{array}{ll} \lfloor \frac{p_{upper}-\beta }{\alpha }+0.5\rfloor ,\; & \alpha >0\\ \lfloor \frac{p_{lower}-\beta }{\alpha }+0.5\rfloor , & \alpha <0 \end{array}\right.$$

Subsequently, we have the difference $t_{diff}$ between the current sampling and estimated switching moments,

(13) $$t_{diff}=t_{pre}-t_{cur}$$

Here, $t_{cur}$ denotes the current sampling moment.

For purpose of efficient switching, the discriminative criterion of data gathering mode is developed based on the difference between the current sampling and estimated switching moments. Suppose we have the discriminative threshold $t_{tol}$ for the switching moment, the data gathering modes is determined according to the following two conditions. 

(a) If $t_{diff}>0$ and $t_{diff}<t_{tol}$, the sensor node notifies the Sink of implementing data reconstruction after conducting $t_{pre}$-th sampling. Subsequently, the prediction-based mode is triggered with the original sampling interval *T*. Otherwise, the CS continues undertaking the task of data gathering.

(b) If $t_{diff}<0$ and $\left|t_{diff}\right|<t_{tol}$, the sensor node immediately notifies the Sink of implementing data reconstruction. Subsequently, the prediction-based mode is triggered with the original sampling interval *T*. Otherwise, the CS continues undertaking the task of data gathering.

**Figure 3:**
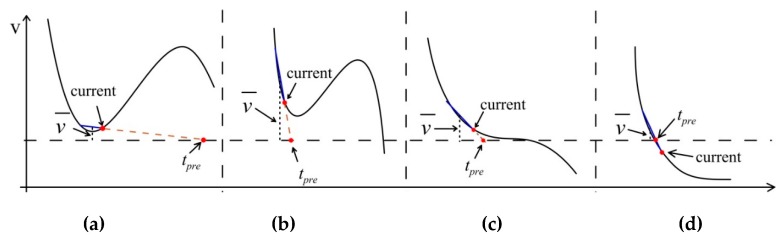
Typical discriminative cases from compressed sensing (CS) to prediction-based modes: (**a**) Wrong switching behavior resulting from inappropriate discriminative threshold for the switching moment; (**b**) wrong switching behavior resulting from inappropriate discriminative threshold for the difference between the average of most recent data points and the prior thresholds; (**c**) actual switching behavior earlier than expected; (**d**) actual switching behavior later than expected.

The two discriminative thresholds, $v_{tol}$ for the sampling values and $t_{tol}$ for the switching moment, are the key to determining the switching behavior of the proposed mechanism. As a matter of fact, it is apparent that the greater discriminative thresholds might result in loose switching condition, which makes the switches become too sensitive. In contrast, too smaller thresholds have potential in resulting in too tight switching condition, which may cause that some switches are missing. Figure 3 illustrates four typically discriminative cases from CS to prediction-based modes, where *current* denotes the current sampling sequence. They can be classified into two categories for making description clear. Figure 3a,b belong to one category, where the wrong judgments are incurred. In Figure 3a, the wrong switching behavior may happen resulting from the inappropriate $t_{tol}$, because the data trace is still above the threshold. On the contrary, Figure 3b shows that the inappropriate $v_{tol}$ might lead to another wrong switching behavior. Additionally, even if the threshold works well so that the wrong switching action can be effectively avoided, it might happen that the actual switching behavior comes later or earlier than expected. Figure 3c,d belong to this category. It has no doubt that this kind of behavior might be dependent on the data characteristics to some degree. 

As we know, it is inevitable that there are fluctuations along the monitored data trace when performing data gathering. It inspires us that the deviation tolerance can be used as one effective tool to develop the discriminative threshold $v_{tol}$. Here, the threshold for the data values $v_{tol}$ is defined as

(14) $$v_{tol}=\gamma \times PDT_{avg}$$

where γ is one adjustment factor; usually, it is greater than one so that it offers some extra margin in addition to the deviation tolerance.

As for the discriminative threshold of switching moment $t_{tol}$, considering the case in Figure 3a, a reduced number of training data points for building the estimation model of switching moments may lead to the decrease in the difference between the average of training data points and the prior threshold. This leads to the fact that the criteria, $v_{diff}$ < $v_{tol}$, tends to be satisfied with high probability. Therefore, as the number of training data points increases, it would be better for avoiding the unnecessary switching behaviors if we have one stricter constraint, that is, smaller discriminative threshold of switching moment. Hence, the threshold of switching moment should be in proportion to the number of training data points. In addition, the average of training data points tends to decrease with an increasing sampling rate, η. Hence, based on a similar analysis, the discriminative threshold of switching moment should be inversely proportional to the sampling rate, η. As a result, the moment threshold for data acquisition is defined as

(15) $$t_{tol}=\left\lfloor \mu \times \frac{m}{\eta }\right\rfloor$$

where *m* denotes the number of training data points for building the estimation model of switching moments, η denotes sparse sampling rate, and μ is the adjustment factor. 

It is worthwhile mentioning that the proposed switching mechanism is developed taking into account sampling rate, the number of training data points and the characteristics of historical data; this definitely enables it to be flexible. More specifically, the proposed switching mechanism can be applied to the applications with the reversed critical and non-critical zones shown in Figure 2. The required adjustment lies in only exchanging the discriminative conditions in Formula (12) for conducting switching from prediction-based to CS modes. Moreover, it is suitable for the applications with one or multiple prior thresholds (only slight adjustment is required). In the case of single prior threshold, one can simply set one of two prior thresholds as infinity according to the specific applications. In the case of multiple prior thresholds, the switching cases can be separated into the multiple cases with single or double prior thresholds no matter how many prior thresholds there are in applications.

## 4. Results and Discussion

In order to evaluate the proposed scheme, two sets of data were used. The data were collected in a realistic nursery green house, at the time interval of 5 min, by means of data logger, developed using Raspberry Pi 3B+ [33], with sensors of air humidity and soil moisture (Leishen Electronics Co. Ltd., Shijiazhuang, China). There was significant difference in the characteristics of the two datasets. The air humidity followed one periodical change trend. On the other hand, the soil moisture changed with the irrigation schedule. The two sets of data were collected from 11 June to 24 June 2018, amounting to 4032 sampling points for each data set. 

Regarding the experimental data, there are some basic environmental requirements for the nursery of tea tree. For the air humidity, double prior thresholds are needed. Particularly, $p_{upper}$ and $p_{lower}$ were set as 80% and 60%, respectively, when considering the data acquisition of air humidity, because air humidity of 60–80% is one requirement for the nursery of tea tree [34]. For soil moisture, a single prior threshold was needed because above 32% of soil moisture is required. As a result, $p_{lower}$ was set to 32% when considering the data acquisition of soil moisture. The experimental parameters are list in Table 1. 

The proposed scheme is evaluated in terms of switching performance and compression ratio. The former is firstly to present how to determine the sampling rate and adjustment factors in (14) and (15), then to investigate the switching behavior in three scenarios where the switching actions occur punctually, as expected, or either earlier or later than expected. The latter involves data compression and quality of data acquisition when data are collected through the integration of CS and various prediction-based approaches. Here, the data compression ratio is the equivalent of energy consumption, since the amount of data traffic has a positive correlation with energy consumption required. Therefore, no energy consumption is explicitly evaluated taking into account routing and wireless media access control protocols. Nevertheless, the proposed data acquisition scheme is not dependent on routing and wireless medium access protocols, which means that it could be directly applied into WSNs operating with any routing and wireless medium access protocols.

### 4.1. Switching Performance

#### 4.1.1. Determination of Parameter Values

It is apparent that the specific values of three parameters, namely, sampling rate, two adjustment factors in (14) and (15), are dependent on the monitored data in applications. In the following, the determination procedure of parameter values is presented as general methodology for various applications. Here, both datasets of air humidity and soil moisture are evenly divided into two parts for further validating the practical applicability of the proposed switching mechanism. That is to say, the specific values of these aforementioned three parameters are determined based on the first half data sets of air humidity and soil moisture. The switching performance is evaluated based on the latter half of the datasets for air humidity and soil moisture. 

Firstly, in order to determine the values of two adjustment factors, the switching behaviors between two data gathering modes are preliminarily investigated with different sparse sampling rates, based on the first half of the datasets for air humidity and soil moisture. It was found that the switching behavior exhibits well when γ and μ are equal to about 1.54 and 0.57, respectively, in the case of air humidity, and γ and μ are equal to about 1.57 and 0.57, respectively, in the case of soil moisture.

Subsequently, the appropriate sampling rate should be determined by considering the switching performance and data reconstruction quality. Here, the reconstruction SNR (Signal-Noise-Ratio) was utilized for evaluating reconstruction quality because it is one of the key factors for choosing appropriate sampling rates in practical application. For the purpose of quantifying the switching performance, the switching perception delay was estimated by averaging the difference of expected and actual switching moments for all mode alternations. For each mode switching from CS to prediction-based modes, the time difference was recorded as positive when actual switching behavior occurred later than expected. On the contrary, the time difference was recorded as negative when the opposite phenomenon occurred. Here, the change in the average delay of switching perception and reconstruction SNR versus sampling rate was chosen to determine the sampling rate.

**Figure 4:**
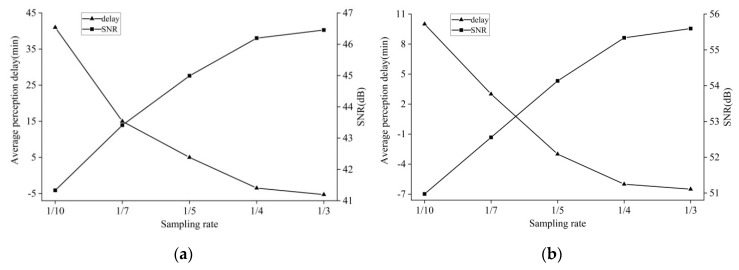
Average switching perception delay and SNR (signal-noise ratio) vs. sampling rate. Positive switching perception delay indicates that actual switching behavior in general occurs later than expected. Negative delay indicates the opposite phenomenon occurs: (**a**) Average switching perception delay and SNR in the case of air humidity; (**b**) average switching perception delay and SNR in the case of soil moisture.

As shown in Figure 4, the average perception delay tends to dramatically decrease with an increasing sampling rate. This is reasonable, because the lower sampling rate would lead to the sparser distribution of sampling data points, which further results in the degradation of predicting switching moments. On the other hand, more data points can contribute to an improvement in determining the switching moments with the increase in the sampling rate. Meanwhile, it can be observed that the greater sampling rate contributes to the improvement in reconstruction SNR. Additionally, it can be observed that there is a narrower range in switching perception delay in the case of soil moisture compared with air humidity, resulting from the difference in data characteristics. Nevertheless, from Figure 4a,b, it can be seen that the proposed scheme can offer reasonable switching moments and reconstruction accuracy when the sampling rate is not less than 1/5, for datasets of both air humidity and soil moisture. Moreover, as the sampling rate increases, the average occurrence moments of switching behaviors tend to come earlier. This is because there are few switching behaviors occurring earlier than before when the sampling rate increases. However, on average, the actual switching moments that come earlier than expected are within the range of one original sampling interval even with the high sampling rate of 1/3.

#### 4.1.2. Investigation of Switching Behavior

**Figure 5:**
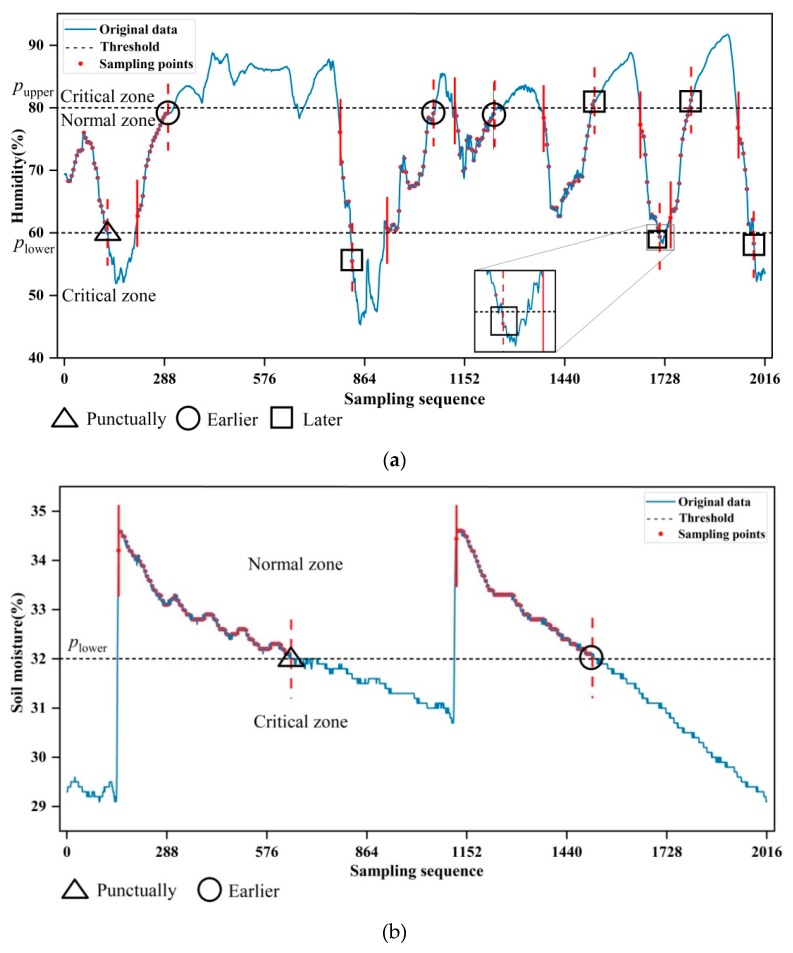
Switching between CS and prediction-based data gathering modes. The CS works with the sampling rate of 1/5: (**a**) Switching behaviors in the case of air humidity. (**b**) Switching behaviors in the case of soil moisture.

Figure 5 shows the switching behaviors between CS and prediction-based data gathering modes. The data points marked by dotted red lines represent the switching behavior from CS to prediction-based modes. By contrast, the data points marked by solid red lines represent the opposite switching behaviors. The triangular icons indicate that the switching behaviors occur punctually, as expected. The circular and square icons show that the switching actions occur earlier or later than expected.

As illustrated in Figure 5a, the switching events occur seventeen times along with the data trace of air humidity when the sampling rate is equal to 1/5. It can be seen that the CS is triggered for sparsely sampled non-critical data points from 10 to 126 of the original sequence, and then the prediction-based mode is triggered for gathering the original data with the sequence numbers of 127 to 210. Subsequently, the CS is again triggered in place of prediction-based mode for data gathering, and so on. Overall, there are nine switching events from prediction-based back to CS modes. By contrast, the opposite switching behaviors occur ten times. It can be seen that all switches from prediction-based to CS modes occurred later than expected. This resulted from our intention that the switching mechanism should be tolerant to the data fluctuation around the threshold. Regarding nine switching behavior events from CS to prediction-based modes, it is witnessed that the switches came earlier than expected at the three data points marked by circular icons, where there are dense data points close to the threshold. This shows that the proposed switching mechanism has good potential to deal with the gathering issues of critical data. By contrast, the switching behaviors come later than expected at the five data points marked by square icons, where the data trace goes sharply. The postponed switching behaviors are acceptable because most of them are far less than one sparse sampling interval. In this regard, the propose mechanism is superior to the existing work, which will be demonstrated in the following paragraphs. At the 126-th data points, it occurs punctually, marked by a triangular icon. Based on the aforementioned nine switching events from CS to prediction-based modes, it is found that the switching behaviors occur, on average, about five minutes later than expected, which is equal to the original sampling interval. Therefore, it can be concluded that all switching behaviors are triggered at the appropriate moments.

Figure 5b shows that the switching behavior occurs four times along with the data trace of soil moisture. In total, two switch behaviors from CS to prediction-based modes are triggered at the data points with the sequence numbers of 645 and 1511. It can be seen that the two switching behaviors occur at the exact moment or a little bit earlier than expected. This phenomenon should be ascribed to the proposed adaptive switch mechanism. In particular, with the help of the appropriate threshold of time tolerance, the wrong and premature switching behaviors are effectively avoided by the proposed mechanism at the aforementioned two switching points of soil moisture. 

**Figure 6:**
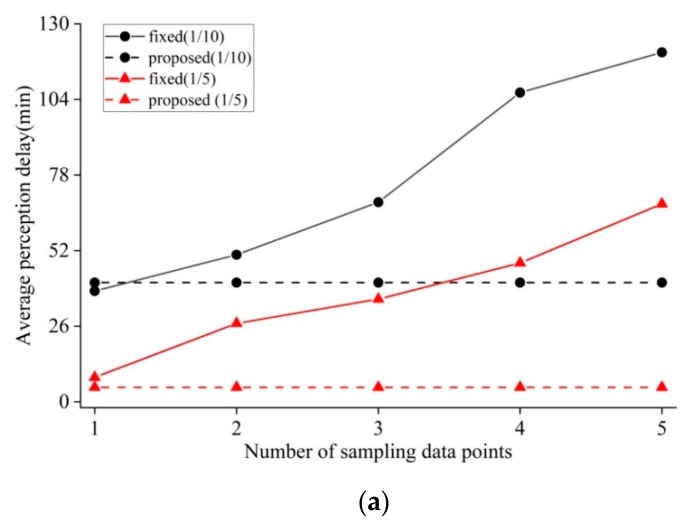

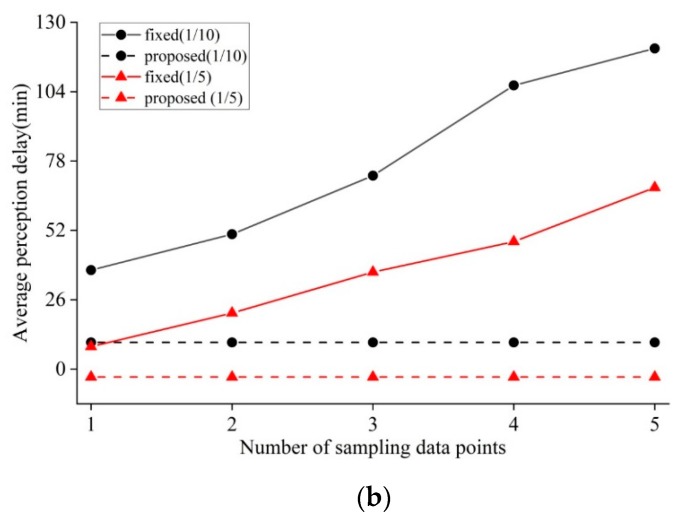
Comparisons of average switching perception delay vs. number of sampling data points: (**a**) Comparison in the case of air humidity; (**b**) comparison in the case of soil moisture.

One might guess that maybe it is good enough to determine the moments of triggering switching action by checking if there are several continuous data points deviating from the predefined threshold (called fixed number of data points, or fixed-point strategy for short). It should be emphasized that the sampling points are sparsely distributed because of implementing CS. Figure 6 shows the comparison of switching perception delay between the proposed switching mechanism and fixed-point strategy with sampling rates of 1/10 and 1/5. Apparently, the switching perception delay of the proposed mechanism remains the same because it relies on the sampling rate and the number of recent data points shown in Formulas (9)–(15); however, this is independent of the number of sparse sampling points mentioned here. As shown in Figure 6, the switching delay, when using fixed point strategy, increases rapidly, and is significantly greater than what the proposed mechanism has. For the two datasets of air humidity and soil moisture, when the number of continuous sampling points is equal to one, the two switching methods have a close approximate perception delay. However, in this case, it is prone to resulting in the frequent, but unnecessary, mode alternations. On the other hand, if the large number of continuous sampling points would help in avoiding the frequent mode alternations, this would be at the expense of high switching perception delay. Therefore, the proposed mechanism can adaptively offer a better switching service of the data gathering modes compared with the existing work.

### 4.2. Compression Ratio and Data Quality

In this section, the proposed scheme integrated with various prediction-based approaches is evaluated in terms of compression ratio and SNR. More specifically, two compression metrics used to evaluate our scheme are defined as

(16) $$CR_{-oh}=\left(1-\frac{A_{trans\_data}}{A_{total\_data}}\right)*100\%$$

(17) $$CR_{+oh}=\left(1-\frac{A_{trans\_data}+A_{overhead}}{A_{total\_data}}\right)*100\%$$

where $CR_{-oh}$ denotes the compression ratio when only data are considered. In contrast, when computing total compression ratio $CR_{+oh}$, in addition to data compression ratio denoted by $CR_{-oh}$, the communication overhead of model synchronization is taken into consideration. That is to say, the total compression ratio is equal to the ratio between the total amount of extra communication overhead and transmitted data to the total amount of original uncompressed data. Note that the extra communication overhead is mainly from the inherent model synchronization in prediction-based approaches.

As mentioned before, there are a few candidate approaches for performing data prediction and packet transmission reduction [27,28,29,30,31,32]. In order to effectively demonstrate the performance of the proposed scheme, three typical data prediction models were chosen as the integrated prediction-based approaches from the three classes: time series modeling, regression methods, and machine-learning techniques.

Taking the model complexity and prediction ability into account, the following three data prediction models were chosen for proceeding with the performance evaluation: DBP, ARIMA, and SVR. The experimental conclusions should have enough potential of offering suggestions for future integration of prediction-based approaches into data acquisition schemes. The extra communication overhead for conducting model synchronization is dependent on the characteristics of both monitored data and data prediction models. Table 2 lists the content and communication amount required when conducting model synchronization of DBP, ARIMA and SVR once, which results from the preliminary experiments.

**Figure 7:**
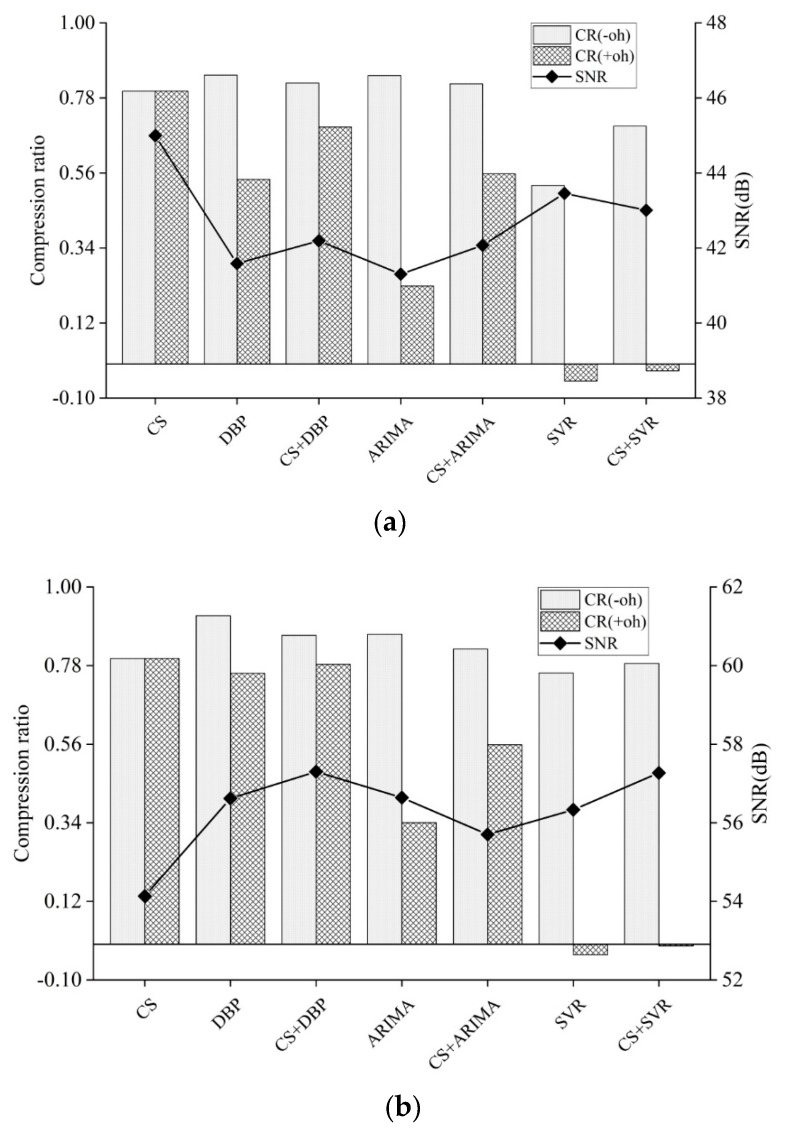
Compression ratio and gathered data quality under various data acquisition schemes. These schemes are from the integration of CS with the sampling rate of 1/5 and various prediction-based approaches: (**a**) Compression ratio and SNR (Signal-Noise-Ratio) in the case of air humidity. (**b**) Compression ratio and SNR in the case of soil moisture.

Figure 7 shows the performance comparison of the integration scheme with CS and several prediction-based approaches, in the case of air humidity and soil moisture. To ease the presentation, the symbol “CS+” is used to represent the proposed scheme integrated with specific prediction model. For example, “CS+DBP” denotes the combination of original CS and DBP. The sampling rate of CS is fixed at 1/5. The symbols $CR_{-oh}$ and $CR_{+oh}$ in the legend denote the data and total compression ratio, corresponding to Equations (16) and (17), respectively.

From Figure 7, it can be observed that the compression ratios tend to significantly decrease for the data prediction models of DBP, ARIMA and SVR when the model synchronization overhead is taken into account. By contrast, the CS has the same compression ratio because no model synchronization is required. This explicitly shows that model synchronization causes the increase in the communication overhead. Particularly, in the case of air humidity and soil moisture, the overhead of DBP model synchronization can lead to the decrease in compression ratio by 54% and 17%, respectively. In addition, it is worth mentioning that the overhead of model synchronization in ARIMA and SVR causes a more severe drop in the compression ratio, a negative compression ratio even appears in SVR. Usually, the model synchronization overhead is mainly from the number of model parameters, and the frequency of model synchronization. As tabulated in Table 2, ARIMA has twice as much communication overhead of model synchronization compared with DBP, and SVR has more than six times. During the experiment, it can be seen that ARIMA has slightly more time of model synchronization than DBP, but three times less than SVR. This phenomenon reveals that the data prediction model could not be suitable for WSNs application if it required high model synchronization frequency or communication overhead.

Figure 7 also depicts the gain in compression ratio offered by the combination of CS and prediction-based approaches. It can be observed that the proposed scheme can offer one compression ratio trade-off between CS and prediction-based modes, in the case of both air humidity and soil moisture. Moreover, the proposed scheme is capable of achieving more than a 30% improvement in reducing the sampling points over the original prediction-based approaches. Therefore, it can be seen that the introduction of CS can bring significant improvements for original prediction-based approaches in the energy efficiency of data acquisition. Note that the specific prediction-based approaches do not contribute to the reduction of sampling points because it is mainly determined by specific sampling rate and application thresholds. Furthermore, the CS can usually bring energy efficiency gains when it is integrated with the approaches with more frequency and communication overhead of model synchronization.

From Figure 7, it can be seen that original CS offers the best SNR when gathering air humidity; however, it offers the worst SNR when gathering soil moisture. This is determined by the inherent mechanism and the characteristics of monitored data. Through scrutinizing the SNR, it is found that there is no significant SNR difference among other data acquisition schemes because the maximal SNR deviation is less than 3%. For the purpose of deeply investigating the difference in the quality of gathered data, systematic SNR comparisons are made as follows. First of all, the comparisons are made among three data prediction models. Although it is shown that SVR provides the best SNR when conducting data gathering of air humidity, it is at the expense of a larger amount of data transmission. By contrast, DBP and ARIMA are able to transmit less data with a comparable level of accuracy. In the case of gathering soil moisture, more data transmission does not contribute to the improvement in SNR of SVR. Subsequently, comparisons are made among all data acquisition schemes. It is observed that the integration can provide approximate or even better SNR in comparison with original prediction-based approaches. Therefore, from the simulation experiment of air humidity and soil moisture, we can draw a conclusion that the integration of CS and prediction-based approaches is capable of achieving significant improvement in the energy efficiency over the original prediction-based approaches without loss in accuracy. Additionally, it is shown that a data prediction model with low communication and computation overhead, for example, DBP, is preferable after observing the compression ratio and the quality of data acquisition.

### 4.3. Discussion

Based on the above experimental results, we have the following observations. Compared with the traditional methods, the proposed data acquisition scheme has the characteristics of providing higher compression ratio resulting from CS, as well as more effective sensing of critical events from adaptive switching mechanism and prediction-based approaches. More specifically, with the help of the adaptive switching mechanism, the data gathering modes could be triggered at appropriate moments as the phenomena change so that the proposed data acquisition scheme could offer good ability of the timely sensing of critical events. Thanks to adaptive deviation tolerance and switching mechanism, the proposed scheme has good flexibility to deal with data acquisition in various applications. In summary, the proposed data acquisition scheme is capable of offering good energy efficiency, as well as high-quality sensing of critical events.

In the proposed scheme, it is required to perform line fitting as part of estimating the switching moments of data gathering modes, which incurs a computation overhead for sensor nodes. Generally, there are low requirements of hardware resource when implementing the line fitting with few data points. Through running on Raspberry Pi 3B+, it is found out that it takes about 30 ms to perform line fitting once. Moreover, on average, there are 1.3 times of line fitting for each round of mode switching. Therefore, given that there are in total 11 switching events in our two groups of air humidity and soil moisture data, the total computation overhead is about 429 ms. On the other hand, in [13], since there is lack of one effective switching discriminative mechanism under the constraint of sparse data points, the readings are taken at original time interval for estimating the switching moment of data gathering modes even if using CS. Thus, the sensor nodes have to regularly perform data compression by multiplying the sampling data by the measurement matrix. Running results on Raspberry Pi 3B+ shows that about 1500 ms are needed to complete the data compression of our air humidity and soil moisture datasets. Actually, this matrix multiplication is restricted by the memory and computing power of sensor nodes. Therefore, this proposed scheme outperforms the existing work in terms of sampling frequency, the requirement of the memory and computing power of sensor nodes.

Additionally, it is worthwhile pointing out that the proposed scheme has good flexibility and scalability. As mentioned in Section 3.3, the proposed data acquisition scheme is suitable for the applications with one or multiple prior thresholds, though it is developed in the case of double prior thresholds. Moreover, due to the inherently adaptive deviation tolerance and switching mechanism, the proposed scheme can be directly applied to handle data acquisition in various applications. Since it is fully distributed, where each node autonomously takes a decision about the way of data acquisition according to the change trend of monitored factors, the proposed schemes can be applied at different network scales for scheduling the data acquisition. One method is that each sensor node autonomously schedules the data gathering modes. The second method is that, for cluster-based networks, the nodes in the same cluster jointly determine their data gathering modes. The first method needs no extra communication overhead. The second method restricts the coordination for the switching of the data gathering modes to be within a cluster. This offers potential, at the expense of a light communication overhead, for further suppressing data transmission by considering cluster-based routing.

## 5. Conclusions

One adaptive data acquisition scheme is developed with the integration of compressed sensing and prediction-based approaches, in which the former and latter are in charge of the acquisition of non-critical and critical data, respectively. With the help of an integration framework, adaptive deviation tolerance, and adaptive switching mechanism, the proposed scheme has good flexibility and scalability to handle data gathering in different applications. Moreover, the data gathering modes could be triggered at appropriate moments when phenomena change, which results in good energy efficiency and high-quality sensing of critical events. Additionally, the lightweight data prediction models are preferable because of the limited in-node resources and communication capability. Future work will focus on the scenarios with outliers arising from sensor failure or lossy wireless links.

## Figures and Tables

**Table 1:** Experimental parameters: *T* denotes original sampling time interval; θ is the significance level; *m* denotes the number of training data points for building the estimation model of switching moments; and η denotes the sparse sampling rate, where 1/10 indicates the sampling interval increases to 10 times original interval, and so on.

Parameters	Value
*T*	5
θ	0.05
*m*	3
η	1/10,1/7,1/5,1/4,1/3

**Table 2:** Content and data amount for model synchronization.

Models	DBP	ARIMA	SVR
Content	α,β	$\phi_{i},\theta_{j}$	ω,b
Amount(Byte)	8	16	50
